# YAP1-dependent regulation of cell size in BCAM-positive limbal corneal progenitor cells

**DOI:** 10.1016/j.exer.2026.111059

**Published:** 2026-05-13

**Authors:** Kosei Suzuki, Shinri Sato, Markus H. Frank, Natasha Y. Frank, Yuzuru Sasamoto

**Affiliations:** a Department of Ophthalmology, University of Washington, Seattle, WA, USA; b Transplant Research Program, Boston Children's Hospital, Boston, MA, USA; c Harvard Stem Cell Institute, Harvard University, Cambridge, MA, USA; d Harvard Skin Disease Research Center, Department of Dermatology, Brigham and Women's Hospital, Boston, MA, USA; e School of Medical and Health Sciences, Edith Cowan University, Perth, Western Australia, Australia; f Division of Genetics, Brigham and Women's Hospital, Boston, MA, USA; g Department of Medicine, VA Boston Healthcare System, Boston, MA, USA

**Keywords:** ABCB5, BCAM, YAP1, Cell size, Limbal stem cell, Transit-amplifying cells

## Abstract

Cell size has been associated with stem and progenitor cell states across multiple tissues, yet its regulation in the human corneal epithelium remains incompletely understood. This study aimed to characterize cell-size differences among limbal and corneal epithelial cell populations defined by the limbal stem cell (LSC) marker ABCB5 and the transit-amplifying cell (TAC) marker BCAM, and to investigate the molecular regulators of progenitor cell size. ABCB5-positive LSCs and BCAM-positive TACs were identified and isolated by flow cytometry, and the mean cell size was estimated using forward scatter (FSC). As a result, ABCB5-positive LSCs were significantly smaller than ABCB5-negative limbal epithelial cells, and BCAM-positive TACs exhibited a smaller cell size than BCAM-negative cells, particularly in the limbus. Immunofluorescence staining showed that Yes-associated protein 1 (YAP1) and BCAM were co-expressed in basal epithelial cells in the human limbus and cornea. Expression of the YAP1 transcriptional target gene *CYR61* was elevated in BCAM-positive limbal cells. SiRNA-mediated knockdown of *YAP1* or *BCAM* in cultured human limbal epithelial cells resulted in a significant increase in cell size. These findings identify small cell size as a characteristic feature of limbal BCAM-positive TACs and demonstrate that progenitor cell size is regulated by YAP1 and BCAM, highlighting an interaction between physical cell properties and progenitor function in the corneal epithelium.

## 1. Introduction

The continuous turnover of corneal epithelium is maintained by limbal stem cells (LSCs) occupying the basal epithelial layer of the limbus, a specialized transitional zone between the cornea and conjunctiva (Davanger and Evensen, 1971;Schermer et al., 1986;Kenyon and Tseng, 1989). LSCs give rise to transit-amplifying cells (TACs), which undergo rapid proliferation and migrate centripetally and superficially to replenish the differentiated layers of the corneal epithelium (Beebe and Masters, 1996;Castro-Muñozledo and Gómez-Flores, 2011;Park et al., 2019;Thoft and Friend, 1983;Yoon et al., 2014). Based on differentiation status and anatomical location, TACs are further subdivided into early TACs in the basal limbus and late TACs in the basal central cornea, with late TACs exhibiting reduced proliferative capacity and increased expression of differentiation markers such as keratin 12 (Kaplan et al., 2019;Lehrer et al., 1998).

The prospective identification of discrete epithelial progenitor populations has enabled a more precise investigation of the corneal epithelial hierarchy and its regulation. We previously identified ATP-binding cassette subfamily B member 5 (ABCB5) as a functional cell surface marker of human LSCs (Ksander et al., 2014). More recently, we demonstrated that basal cell adhesion molecule (BCAM) marks a proliferative TAC population with robust colony-forming capacity and corneal differentiation potential (Sasamoto et al., 2022, 2023). These markers enable the isolation of LSCs and TACs by flow cytometry, providing a powerful platform for interrogating cellular and molecular features associated with distinct progenitor states.

Cell size has emerged as an important physical parameter linked to stem and progenitor cell function across multiple tissues, with undifferentiated cells generally exhibiting smaller size than their differentiated counterparts (Li et al., 2015). Experimental evidence indicates that maintenance of small cell size is critical for preserving stem cell potential, including in hematopoietic stem cells and epidermal progenitors (Lengefeld et al., 2021;Zijl et al., 2025). In the corneal epithelium, basal epithelial cells are smaller than suprabasal cells, and limbal epithelial cells are smaller than those in the central cornea (Romano et al., 2003), suggesting a relationship between cell size and epithelial hierarchy. However, whether reduced cell size is a distinctive feature of molecularly defined LSCs and TACs, and how progenitor cell size is regulated at the mechanistic level, remains poorly understood.

In this study, we demonstrate that limbal ABCB5-positive LSCs and BCAM-positive TACs are characterized by significantly smaller cell size compared with other corneal epithelial populations. Furthermore, we identify the Hippo pathway effector Yes-associated protein 1 (YAP1) as a key regulator of cell size in BCAM-positive TACs. These findings establish cell size as a distinguishing physical property of corneal epithelial progenitors and provide new insight into how molecular signaling pathways intersect with physical cell state to regulate progenitor function within the limbal epithelium.

## 2. Material and methods

### 2.1. Human cornea dissection

Human corneas were obtained from the eye banks, Saving Sight (Kansas City, MO) and CorneaGen (Seattle, WA) under the Institutional Review Board (IRB) approval. Dissection was performed as previously reported (Sasamoto et al., 2020, 2023). Briefly, 8 mm central corneal buttons were harvested using a disposable biopsy punch (Integra Life-Sciences, Plainsboro, NJ), followed by the removal of corneal endothelium. Limbal and central corneas were digested with PluriSTEM Dispase II Solution (MilliporeSigma, Burlington, MA) for 1 h at 37 °C. Following trypsinization, the cells were either processed for flow cytometric analysis or cultured for *in vitro* expansion. The culture medium consisted of DMEM/F12 medium (Thermo Fisher Scientific, Waltham, MA) supplemented with 10 μM Y-27632 (Tocris Bioscience, Bristol, UK), 10 ng/mL keratinocyte growth factor (KGF; PeproTech, Rocky Hill, NJ), and B-27 Supplement (Thermo Fisher Scientific) (Miyashita et al., 2013).

### 2.2. Flow cytometry

ABCB5-positive LSCs and BCAM-positive TACs were isolated as previously described (Sasamoto et al., 2020, 2022, 2023). Dissociated limbal or central corneal epithelial cells were resuspended in a buffer of phosphate-buffered saline (PBS) (GE Healthcare Life Sciences, Marlborough, MA) with 2% fetal bovine serum (FBS) (Thermo Fisher Scientific). Cells were incubated with antibodies for 30 min on ice. Antibodies used were: 2.5 μg/ml mouse anti-ABCB5 monoclonal antibody (mAb) clone 3C2–1D12 (Frank et al., 2003;Ksander et al., 2014) conjugated with Alexa Fluor 647 (Thermo Fisher Scientific), 0.5 μg/ml Brilliant Violet 421-conjugated anti-CD45 mAb (clone 2D1, BioLegend, San Diego, CA), and 4 μg/ml VioBright FITC-conjugated anti-BCAM mAb (Miltenyi Biotec, Bergisch Gladbach, Germany). CD45 staining was used to exclude hematopoietic, and Propidium Iodide Staining Solution was used to identify and exclude dead cells. Cell sorting and analysis were performed using a FACSAria II cell sorter (BD Biosciences, San Jose, CA), and obtained data were analyzed with FlowJo v10.6.1 (BD Biosciences). Mean cell size was estimated using forward scatter (FSC), which is proportional to cell size. A 5% margin was applied between positive and negative populations for ABCB5 or BCAM expression.

### 2.3. Immunofluorescence staining

Human whole globes were fixed overnight at 4 °C in 10% neutral buffered formalin (Fisher Scientific, Pittsburgh, PA) and then transferred to 70% ethanol. Tissues were embedded in paraffin and sectioned at a thickness of 5 μm. The sections were then deparaffinized, and antigen retrieval was carried out following standard protocols. Permeabilization and blocking were carried out for 30 min at room temperature in Tris-buffered saline (TBS; Boston BioProducts, Ashland, MA) containing 0.3% Triton X-100 (MilliporeSigma) and 5% normal donkey serum (Jackson ImmunoResearch Laboratories, West Grove, PA). Sections were incubated overnight at 4 °C with mouse anti-YAP mAb (1:100, Santa Cruz Biotechnology, Dallas, TX) and rabbit anti-BCAM polyclonal antibody (pAb) (1:100, NOVUS Biologicals, Centennial, CO). After TBS washes, the sections were incubated for 1 h at room temperature with Alexa Fluor 488-conjugated mouse secondary antibody (Abcam, Cambridge, UK) and Alexa Fluor 568-conjugated rabbit secondary antibody (Abcam). Nuclei were counterstained with Hoechst 33342 (Thermo Fisher Scientific) for 10 min. Slides were coverslipped using ProLong Gold Antifade Mountant (Thermo Fisher Scientific). Images were acquired using a Nikon C2+ confocal microscope and analyzed by NIS-Elements AR v4.30.01 (Nikon, Tokyo, Japan).

### 2.4. RNA extraction, reverse transcription and quantitative PCR (qPCR)

Total RNA was isolated using the RNeasy Plus Mini Kit (QIAGEN, Hilden, Germany). Residual genomic DNA was eliminated with the DNA-free^™^ DNA Removal Kit (Thermo Fisher Scientific). cDNA synthesis was carried out with the High-Capacity cDNA Reverse Transcription Kit (Thermo Fisher Scientific). qPCR was performed using TaqMan^™^ Fast Universal PCR Master Mix (Thermo Fisher Scientific) and TaqMan^™^ Gene Expression Assay probes (Thermo Fisher Scientific). TaqMan^™^ probes used were *GAPDH* (Hs99999905_m1) and *CYR61* (Hs00155479_m1). qPCR reactions were performed on the StepOne-Plus^™^ Real-Time PCR System (Thermo Fisher Scientific) under the following conditions: 95 °C for 20 s, followed by 50 cycles of 95 °C for 1 s and 60 °C for 20 s. ΔΔCt was calculated using GAPDH as the reference gene.

### 2.5. RNA interference

siRNA-mediated gene knockdown (KD) was performed following previously described protocols (Fujimoto et al., 2019). Briefly, siRNA transfection was carried out immediately after cell passage using Lipofectamine^™^ RNAiMAX Transfection Reagent (Thermo Fisher Scientific). This study utilized Silencer Select siRNAs (Thermo Fisher Scientific), including Silencer^™^ Select Negative Control No.1 siRNA, *YAP1* siRNAs (s20366 and s20368) and *BCAM* siRNAs (s8336 and s8337).

### 2.6. Western blot

Cell lysates were prepared in RIPA buffer (Cell Signaling Technology, Danvers, MA) containing cOmplete^™^ Protease Inhibitor Cocktail (MilliporeSigma). Lysates were combined with SDS-sample buffer (Boston BioProducts) and 2-mercaptoethanol (MilliporeSigma) and heated at 95 °C for 10 min. The denatured proteins were separated by SDS-PAGE (Bio-rad, Hercules, CA) and transferred to PVDF membranes (GE Healthcare Life Sciences, Marlborough, MA). Membranes were blocked with 5% blotting-grade blocker (Bio-Rad) for 1 h at room temperature and incubated overnight at 4 °C with rabbit anti-YAP1 monoclonal antibody (1:5000; Abcam) and rabbit anti-β-actin polyclonal antibody (1:1000; Cell Signaling Technology) diluted in 2.5% blocking buffer. After washing with TBS containing Tween 20 (MilliporeSigma) (TBS-T), membranes were incubated with HRP-conjugated anti-rabbit secondary antibody (1:1000, Cell Signaling Technology) for 1 h at room temperature. Protein band signals were detected using Western Lightning Plus-ECL (PerkinElmer, Waltham, MA) and imaged with a ChemiDoc MP Imaging System (Bio-Rad). Band intensity quantification was performed using Image Lab software v5.2.1 (Bio-Rad) and normalized to β-actin levels.

### 2.7. Colony-forming assay

The colony-forming assay (CFA) was conducted as previously described (Sasamoto et al., 2020, 2022, 2023). Briefly, a total of 500 cultured limbal epithelial cells were seeded onto 6-well plates containing a mitomycin C (MMC) (MilliporeSigma)-treated 3T3-J2 feeder cell layer and subsequently cultured in keratinocyte culture medium (KCM). KCM consists of DMEM without glutamine and Ham's F-12 Nutrient Mix (Thermo Fisher Scientific) combined at 3:1 ratio, supplemented with 10% FBS, 0.5% (vol/vol) insulin transferrin selenium solution (Thermo Fisher Scientific), 0.4 μg/ml hydrocortisone hydrogen succinate (MilliporeSigma), 1 nM cholera toxin (List Biological Laboratories, Campbell, CA), 2 nM 3,3′,5-triiodo-l-thyronine sodium salt (MilliporeSigma), 2.25 μg/ml bovine transferrin HOLO form (Thermo Fisher Scientific), 2 mM L-glutamine (Thermo Fisher Scientific), and 1% (vol/vol) penicillin-streptomycin solution (GE Healthcare Life Sciences). KGF (10 ng/mL) and Y-27632 (10 μM) were added to the media immediately before use. After 10 days of culture, colonies were fixed with 10% neutral buffered formalin and stained using Rhodamine B (MilliporeSigma). Colony-forming efficiency was determined by dividing the number of colonies per well by the number of seeded cells and expressing the result as a percentage. Relative colony-forming efficiency was calculated relative to control samples.

### 2.8. Statistical analyses

Data are expressed as mean ± standard deviation (SD), and all analyses were two-sided. Statistical tests used (paired-t test, Tukey's multiple comparisons test and Dunnett's multiple comparisons test) are specified in the corresponding figure legends. Statistical significance was set at p < 0.05. *p < 0.05, **p < 0.01, ***p < 0.001, ****p < 0.0001.

## 3. Results

### 3.1. ABCB5-positive LSCs and BCAM-positive TACs exhibit smaller cell size

**Fig. 1.**
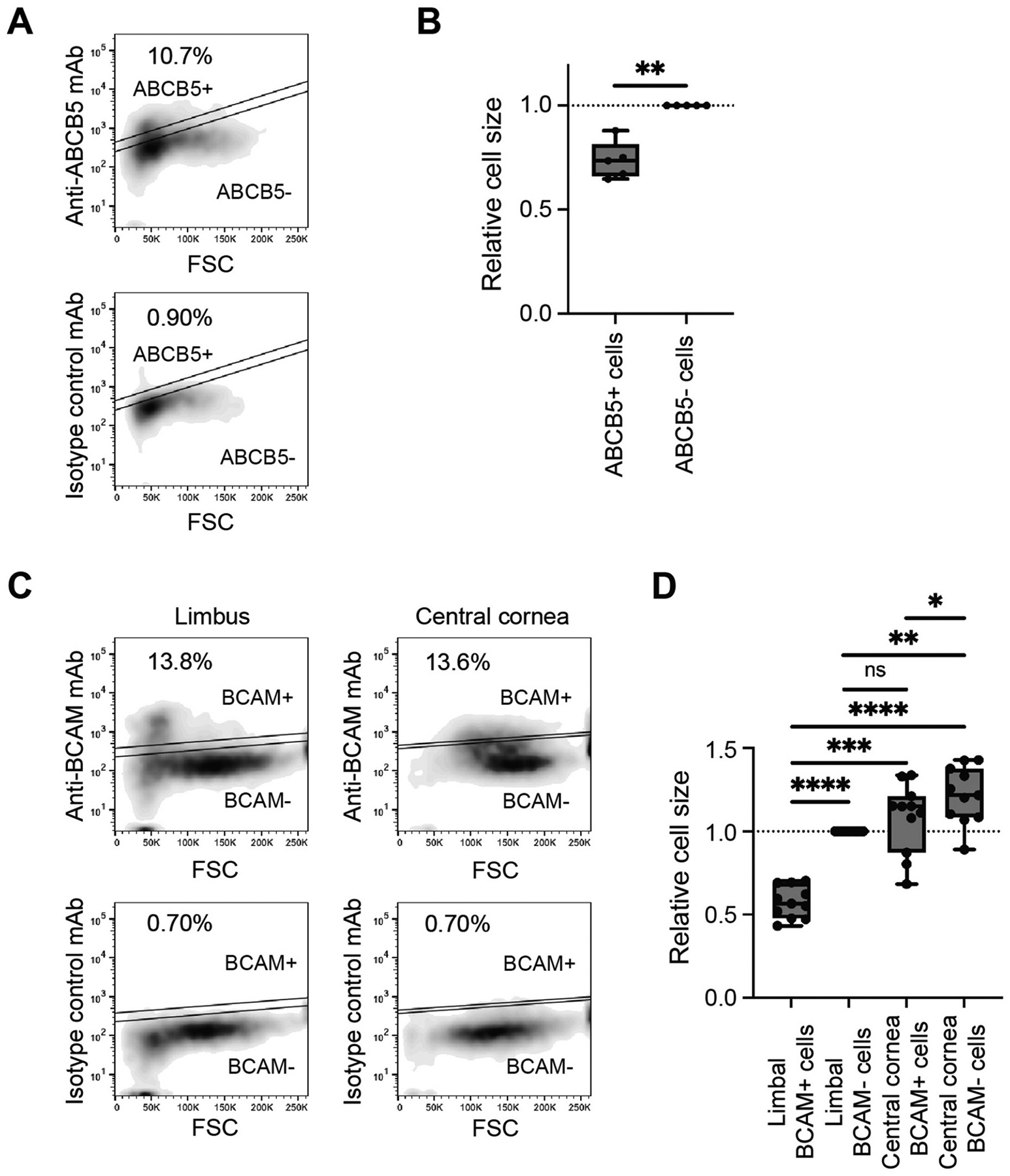
ABCB5-positive LSCs and BCAM-positive TACs exhibit reduced cell size in the limbus. (**A**) Representative flow cytometric gating strategy used to identify ABCB5-positive limbal stem cells (LSCs) and ABCB5-negative limbal epithelial cells. (**B**) Quantitative analysis of cell size, estimated by FSC, demonstrates that ABCB5-positive LSCs are significantly smaller than ABCB5-negative limbal cells (26.4 ± 9.1 %, p = 0.0029, n = 5 donors). (**C**) Representative flow cytometric identification of BCAM-positive transit-amplifying cells (TACs) and BCAM-negative cells in the limbus (left) and central cornea (right). (**D**) BCAM-positive cells in the limbus exhibit a marked reduction in cell size compared with BCAM-negative limbal cells (42.6 ± 9.5% reduction; p < 0.0001; n = 11 donors). BCAM-positive cells in the central cornea are also smaller than their BCAM-negative counterparts (11.3 ± 12.7% reduction; p = 0.0497; n = 11 donors). Comparative regional analysis shows that limbal BCAM-positive cells are significantly smaller than BCAM-positive cells from the central cornea (44.6 ± 14.8% reduction; p = 0.0001; n = 11 donors), and that limbal BCAM-negative cells are modestly but significantly smaller than BCAM-negative cells from the central cornea (16.2 ± 12.7% reduction; p = 0.0078; n = 11 donors). Data are shown as box-and-whisker plots overlaid with individual data points (center line: median; box: interquartile range; whiskers: min–max). *p < 0.05, **p < 0.01, ***p < 0.001, and ****p < 0.0001 by paired *t*-test (**B**) and Tukey's multiple comparisons test (**D**).

Flow cytometric analyses were performed to assess cell size using FSC as a surrogate marker. ABCB5-positive LSCs exhibited significantly smaller size compared with ABCB5-negative limbal epithelial cells (26.4 ± 9.1 %, p = 0.0029, n = 5) (Fig. 1A and B). Similarly, BCAM-positive cells in the limbus were significantly smaller than BCAM-negative limbal cells (42.6 ± 9.5% reduction, p < 0.0001, n = 11), and BCAM-positive cells in the central cornea were also smaller than their BCAM-negative counterparts (11.3 ± 12.7% reduction, p = 0.0497, n = 11) (Fig. 1C and D).

Comparative analyses between anatomical regions further demonstrated that limbal BCAM-positive cells were significantly smaller than BCAM-positive cells from the central cornea (44.6 ± 14.8% reduction, p = 0.0001, n = 11). In addition, limbal BCAM-negative cells were modestly but significantly smaller than BCAM-negative cells from the central cornea (16.2 ± 12.7% reduction, p = 0.0078, n = 11) (Fig. 1D).

### 3.2. YAP1 and BCAM control cell size in BCAM-positive cells

**Fig. 2.**
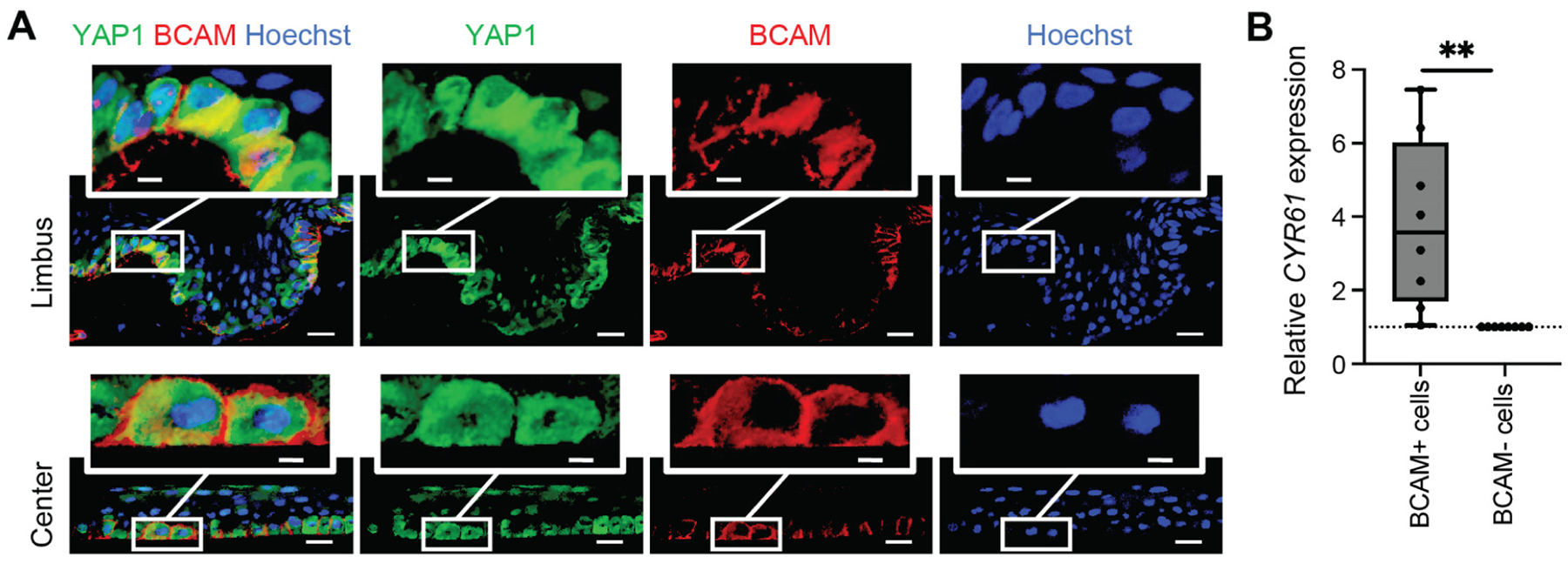
High YAP1 expression in BCAM-positive corneal epithelial cells. (**A**) Representative immunofluorescence staining of human corneal tissue showing co-localization of Yes-associated protein 1 (YAP1; green) and basal cell adhesion molecule (BCAM; red) in basal epithelial cells from the limbus to the central cornea. Nuclei are counterstained with Hoechst 33342 (blue). Scale bar: 20 μm (5 μm for magnified images). Images are representative of three independent donors. (**B**) Quantitative RT-PCR analysis demonstrating significantly higher expression of the YAP1 transcriptional target gene *CYR61* in BCAM-positive limbal epithelial cells compared with BCAM-negative cells (3.83 ± 2.30-fold increase; p = 0.0078; n = 8 donors). Data are shown as box-and-whisker plots overlaid with individual data points (center line: median; box: interquartile range; whiskers: min–max). **p < 0.01 by paired *t*-test.

Yes-associated protein 1 (YAP1), a major downstream effector of the Hippo signaling pathway and a well-established regulator of organ and cell size, has been implicated in progenitor cell expansion across multiple tissues. (Camargo et al., 2007; Huang et al., 2005; Mugahid et al., 2020; Driskill and Pan, 2023). Immunofluorescence analyses revealed both nuclear and cytoplasmic localization of YAP1 in the majority of BCAM-positive cells throughout the limbus and central cornea (Fig. 2A). Consistent with nuclear YAP1 activity, expression of the YAP1 transcriptional target gene *CYR61* was significantly higher in BCAM-positive limbal cells compared with BCAM-negative cells (3.83 ± 2.30-fold increase, p = 0.0078, n = 8) (Fig. 2B).

**Fig. 3.**
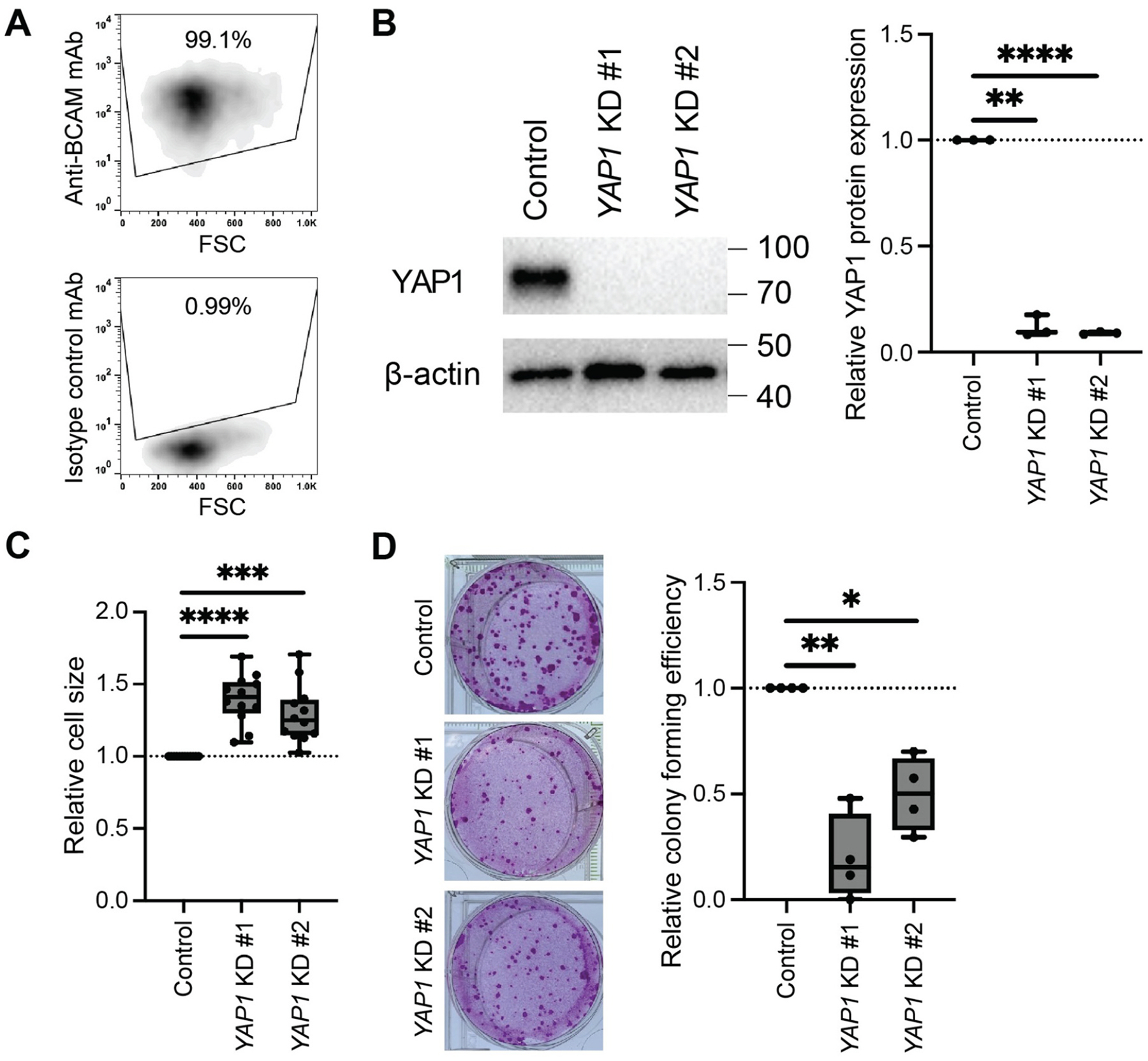
*YAP1* knockdown increases cell size and reduces colony-forming efficiency in limbal epithelial cells. (**A**) Representative flow cytometric analysis of BCAM expression in in vitro-expanded human limbal epithelial cells. Expanded cells maintain high BCAM expression (98.3 ± 0.3%; n = 4 donors). (**B**) Confirmation of *YAP1* knockdown (KD) by Western blot analysis (left). Box-and-whisker plot shows quantitative analysis of YAP1 protein levels normalized to β-actin (n = 3 donors) (right). (**C**) *YAP1* KD using two independent siRNAs (KD#1 and KD#2) results in a significant increase in cell size, as assessed by forward scatter (KD#1: 39.8 ± 17.1% increase, p < 0.0001; KD#2: 29.2 ± 19.8% increase, p = 0.0006; n = 12 donors). (**D**) *YAP1* KD significantly reduces colony-forming efficiency relative to control cells (KD#1: 80.4 ± 20.4% reduction, p = 0.0070; KD#2: 50.1 ± 17.6% reduction, p = 0.0176; n = 4 donors). Data are shown as box-and-whisker plots overlaid with individual data points (center line: median; box: interquartile range; whiskers: min–max). *p < 0.05, **p < 0.01, ***p < 0.001, and ****p < 0.0001 by Dunnett's multiple comparisons test.

**Fig. 4.**
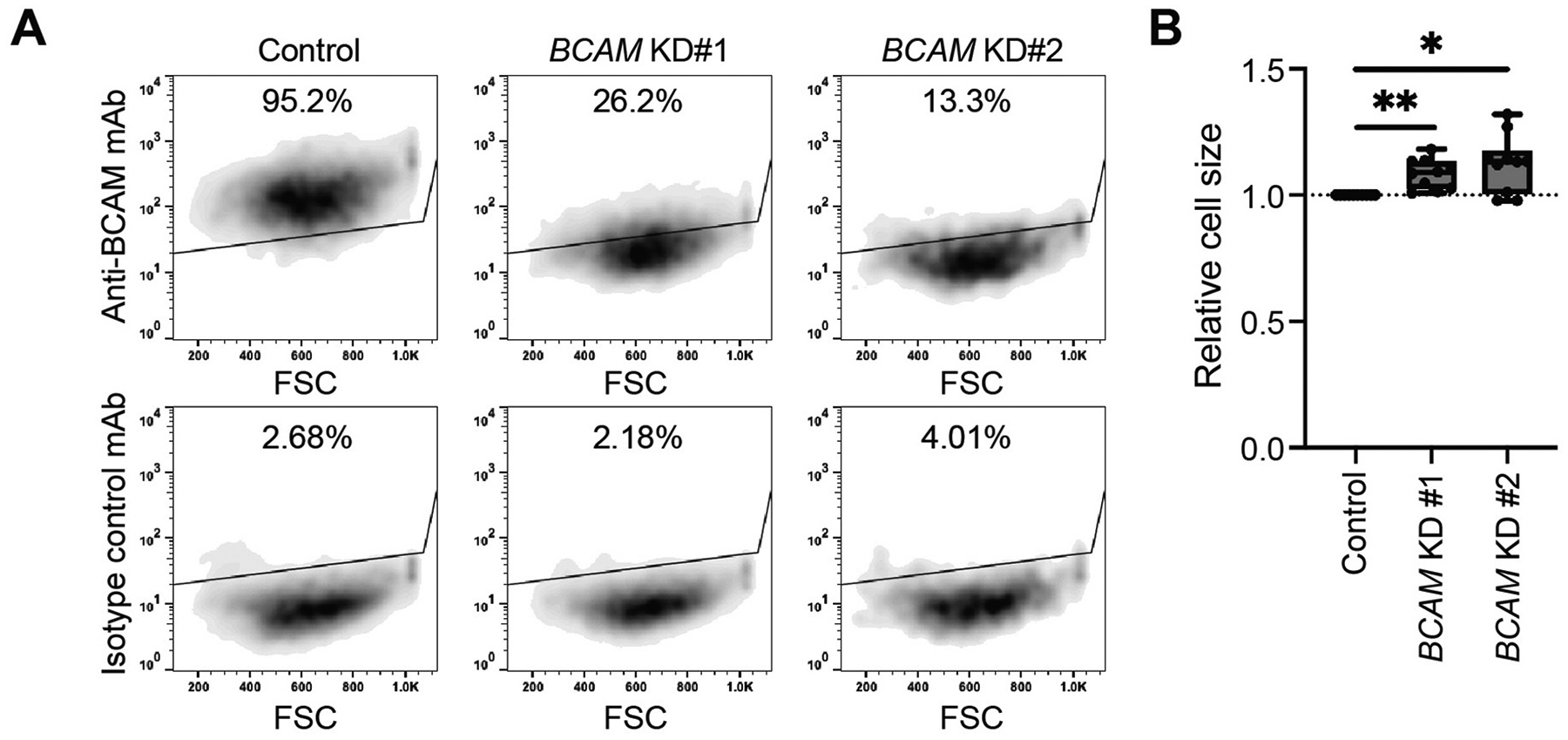
*BCAM* KD increases cell size in limbal epithelial cells. (**A**) Representative flow cytometric profiles showing BCAM expression in control and *BCAM* siRNA-treated human limbal epithelial cells. (**B**) *BCAM* KD using two independent siRNAs (KD#1 and KD#2) results in a significant increase in cell size, as assessed by forward scatter (KD#1: 8.3 ± 5.8% increase, p = 0.0028; KD#2: 12.3 ± 11.4% increase, p = 0.0138; n = 10 donors). Data are shown as box-and-whisker plots overlaid with individual data points (center line: median; box: interquartile range; whiskers: min–max). *p < 0.05 and **p < 0.01 by Dunnett's multiple comparisons test.

*In vitro* expanded limbal epithelial cells retained high BCAM expression (98.3 ± 0.3 %, n = 4) (Fig. 3A) and were therefore used for functional studies. Knockdown of *YAP1* using two independent siRNAs (*YAP1* KD#1 and *YAP1* KD#2) (Fig. 3B) resulted in a significant increase of cell size (*YAP1* KD#1: 39.8 ± 17.1%, p < 0.0001; *YAP1* KD#2: 29.2 ± 19.8 %, p = 0.0006, n = 12) (Fig. 3C). Furthermore, *YAP1* KD significantly reduced colony-forming efficiency (*YAP1* KD#1: 80.4 ± 20.4 %, p = 0.0070; *YAP1* KD#2: 50.1 ± 17.6 %, p = 0.0176, n = 4) (Fig. 3D), indicating a positive role for YAP1 in proliferative capacity. Similarly, *BCAM* KD by two distinct siRNAs, designated as *BCAM* KD#1 and *BCAM* KD#2 (Fig. 4A), led to a modest but significant increase in cell size (*BCAM* KD#1: 8.3 ± 5.8% increase, p = 0.0028; *BCAM* KD#2: 12.3 ± 11.4% increase, p = 0.0138; n = 10) (Fig. 4B).

## 4. Discussion

In this study, we demonstrate that corneal epithelial progenitor populations defined by ABCB5 and BCAM are characterized by significantly smaller cell size compared with their respective negative counterparts, and we identify YAP1 as a key regulator of cell size and proliferative capacity in BCAM-positive TACs. These findings provide new insight into how physical cellular attributes intersect with molecular signaling to regulate progenitor cell function in the human corneal epithelium.

Cell size has emerged as an important determinant of stem and progenitor cell identity across multiple tissues, with smaller size frequently associated with enhanced proliferative potential and reduced differentiation (Dolznig et al., 2004; Li et al., 2015; Rouzaire-Dubois et al., 2004; Tzur et al., 2009). In the corneal epithelium, earlier morphometric studies demonstrated that basal limbal epithelial cells are smaller than cells in the central cornea, suggesting a relationship between cell size and stemness (Romano et al., 2003). By prospectively isolating epithelial subpopulations using ABCB5 and BCAM (Ksander et al., 2014; Sasamoto et al., 2022), our study extends these observations by directly linking reduced cell size to molecularly defined LSCs and TACs. The pronounced size reduction observed in limbal BCAM-positive TACs, compared with both BCAM-negative cells and BCAM-positive cells from the central cornea, supports the concept that early TACs retain progenitor-like physical properties consistent with their high proliferative capacity.

Our data identify YAP1 (Camargo et al., 2007) as a critical regulator of cell size in BCAM-positive progenitor cells. YAP1 showed nuclear and cytoplasmic localization in BCAM-positive basal epithelial cells, accompanied by increased expression of the YAP transcriptional target gene *CYR61*, indicating active transcriptional signaling. Functionally, *YAP1* KD resulted in cell enlargement together with a significant reduction in colony-forming efficiency, supporting a model in which YAP1 maintains a small-cell, highly proliferative progenitor state. These findings are consistent with established roles of YAP signaling in regulating cell growth, proliferation, and tissue homeostasis in epithelial systems (Kasetti et al., 2016; Raghunathan et al., 2014; Bhattacharya et al., 2023) and extend its function to the regulation of cell size in human corneal progenitor cells.

The limbal niche is characterized by distinct biomechanical properties compared with the central cornea, including differences in extra-cellular matrix composition and tissue stiffness (Gouveia et al., 2019). These biomechanical cues regulate limbal epithelial behavior through YAP-dependent mechanotransduction pathways (Gouveia et al., 2019). In this context, the presence of active YAP1 signaling in BCAM-positive progenitors that nevertheless maintain a small cell size suggests a finely balanced integration of biochemical signaling and physical constraints within the limbal microenvironment. Our observation that *BCAM* KD modestly but significantly increased cell size further supports a role for cell–matrix interactions in regulating progenitor cell geometry. As BCAM is a laminin-binding adhesion molecule (Moulson et al., 2001; Kikkawa et al., 2002; Parsons et al., 2001), altered adhesion to the basement membrane may influence cytoskeletal organization and mechanical tension, thereby affecting cell size and YAP activity.

Taken together, these findings support a model in which YAP1 and BCAM cooperatively maintain a progenitor state characterized by small cell size and high proliferative potential in limbal TACs. As cells migrate centripetally toward the central cornea and undergo progressive differentiation, changes in adhesion, mechanical environment, and loss of YAP signaling may contribute to cell enlargement and loss of progenitor features. While this study establishes a functional link between YAP1, BCAM, and cell size regulation, further work integrating single-cell transcriptomic or spatial analyses will be necessary to determine how cell size alterations influence lineage commitment and differentiation trajectories *in vivo*.

In summary, our study identifies small cell size as a defining physical feature of ABCB5-positive limbal stem cells and BCAM-positive TACs and demonstrates that YAP1 and BCAM play central roles in regulating cell size and proliferative capacity in corneal epithelial progenitors. These findings advance our understanding of how physical and molecular mechanisms converge to maintain corneal epithelial homeostasis and provide a foundation for future studies exploring how modulation of progenitor cell size may influence corneal regeneration and repair.

## Data Availability

Data will be made available on request.
